# Back to the Roots of Nursing: Qualitative Study on the Experience of Nurses in the Front Line During the COVID-19 Pandemic

**DOI:** 10.3389/fmed.2022.903517

**Published:** 2022-06-09

**Authors:** Anna De Benedictis, Raffaella Gualandi, Sabrina Saccoccia, Claudio Pensieri, Michela Piredda, Francesco De Micco, Anna Marchetti, Gabriella Facchinetti, Alessia Assunta Pasquarelli, Chiara De Carolis, Irene Di Blasio, Daniela Tartaglini, Rossana Alloni

**Affiliations:** ^1^Campus Bio-Medico University Hospital Foundation, Rome, Italy; ^2^Campus Bio-Medico University of Rome, Rome, Italy; ^3^S.S. Annunziata Hospital, Chieti, Italy; ^4^S. Carlo of Nancy Hospital, Rome, Italy; ^5^Agostino Gemelli University Hospital Foundation, Rome, Italy

**Keywords:** COVID-19, nursing, person centered care, job well done, quality management

## Abstract

The COVID-19 emergency has led many health facilities to reorganize themselves in a very short time to meet the urgent needs for intensive, semi-intensive or ordinary care of SARS-CoV-2 patients. In this pandemic, characterized by speed of transmission and severity of respiratory symptoms, care has been affected by the increase in volume and clinical complexity of patients, the sudden and unpredictable staff decrease and the lack of support from family members / caregivers. At the same time, experience in the field has shown how “informal” resources have been activated, which enabled to treat the highest possible number of patients above the real availability of resources. The purpose of this study was to explore the experiences of nurses involved in frontline care (COVID Centers) during the pandemic with a particular focus on professional motivation and on the development of technical-professional and personal skills. A study with a qualitative research design using focus group technique was conducted. Two focus groups were held with nine nurses. Data were analyzed with inductive content analysis. The findings can be summarized in five main categories: professional identity; motivation and sense of mission; development of professional and personal skills; spirituality; person-centered care; uniqueness of the lived experience. These findings shed new light on the correlation between motivation, professional identity and value, sense of duty and sense of belonging to the professional group. Moreover, the experience in the COVID Centers represented a valuable opportunity for participants to rediscover some specific issues related to nursing professional identity and to develop new personal and technical-professional skills in a very short time. Finally, nurses experienced once again how the nurse-patient relationship and basic care are essential to provide effective and excellent care, even and especially for patients in critical conditions. Nurses re-discovered, in a careful body care and basic care, irreplaceable elements to give back to patients, often dying, their own dignity, and all the needed closeness and attention necessary also to compensate the absence of the loved ones. These elements represent a way to concretely and deeply express the ethics of a job well done in nursing.

## Introduction

The COVID-19 emergency has led many health facilities to reorganize themselves in a very short time to cope with the urgent needs for intensive, semi-intensive or ordinary care of SARS-CoV-2 patients. In this pandemic, characterized by the speed of transmission and severity of respiratory symptoms, patient care has been affected by the pressure of some variables, including the increase in the volume and complexity of patient care, the sudden and unpredictable decrease in staff and the lack of support from family members / caregivers. At the same time, “informal” resources have been activated to treat the highest possible number of patients above the real possibility (1–6).

Recent studies have shown that working experience in emergency situations can have a strong impact on the motivation of the personnel involved. In fact, despite significant efforts, professionals during emergencies show greater resilience, dedication and active cooperation to overcome difficulties and save more lives (3). In many cases, this phenomenon can represent an important lever for change and growth (4). A strong motivation can affect the improvement of performances, and the experience of a health emergency may also have a strong impact on the development of professional and personal skills. Work pressure during an health emergency may push nurses to improve their soft skills, bringing significant collective growth (5) and strengthening their professional identity and value (7). Many of the nurses involved in caring for SARS-CoV-2 patients reported a unique experience in terms of sharing work with other team members and managing new and complex clinical and organizational situations (8, 9). Numerous studies have been carried out to explore the experience of nurses involved in the frontline during the SARS-CoV-2 emergency, but there is still limited literature referring to the possible impact of the pandemic on nurses' motivation, as well as on the development of new skills (9).

## Materials and Methods

### Aims and Objectives

This study aimed to explore the experience of nurses involved in the care of inpatients in a COVID Center of an Italian University Hospital during the first period of pandemic emergency. The main study's objectives were to explore whether and how the pandemic has affected nurses involved in frontline care with a particular focus on professional motivation, on the willingness to care for COVID-19 patients, and in the growth in personal and professional skills.

### Research Design

According with the descriptive aims of the study, a qualitative research design was used (10). Focus group technique was selected as data collection method as it allows generating rich information, also and especially thanks to the interaction between the participants, to understand the experience of nurses during the pandemic era with respect to the research questions (11, 12). Indeed, this technique tends to be more advantageous for the early stages of a research study, as in the case of the study conducted, and it allows to collect more information also through observation and interaction between participants.

Another secondary rationale for choosing the focus group technique is that, at the time of the study, the nurses were still very emotionally impacted by their experience in the COVID centers, and it was thought that the opportunity to share their experiences with other colleagues who had had the same experience-rather than face-to-face with the researchers-might help them express their opinions better and more easily.

### Setting and Timing

The study was carried out at a 380-beds University Hospital in Rome (Italy), which during the first period of the pandemic had two COVID centers with a total availability of 94 beds for COVID-19 patients, including intensive and semi-intensive areas. The study was conducted in the period between December 2020 and June 2021.

### Sampling, Recruitment and Participants

The study participants were nurses who worked in the COVID Centers of the Hospital, they were approached by mail. Purposive sampling was conducted guided by the research questions, in order to obtain the saturation of the topics under study (13, 14). An over recruitment was performed to allow for potential drop out. The initial sample consisted of 20 nurses, 10 from each focus group, but at the time of the study some nurses were unable to participate. It is important to consider that the study was conducted during the pandemic, with all the resulting critical issues related to the need to cover shifts.

### Ethics Issues

The study was approved by the General Management of the University Hospital on November 2020 and by the University Ethics Board on December 2020 (Prot. 106.20 OSS ComEt CBM). Participants were informed about the study aims and procedures, and they were asked to sign informed consent for participation in the study before each focus group. Participant personal data were treated as confidential.

### Data Collection and Analysis

The focus groups were moderated by two facilitators, one of which was a Research Nurse; she also was the principal investigator of the study and acted as the group leader, as recommended (15, 16). The other researcher worked as co-facilitator ensuring that discussions were tape-recorded and observed participants' non-verbal behavior and group dynamics utilizing a specific observation grid.

Opening instructions were provided before starting each discussion. Both groups developed lively, open and spontaneous discussions, sharing a wide range of personal experiences, feelings and opinions. A multidisciplinary panel, composed of middle managers, nursing coordinators and nurses not involved in the focus groups, defined the topic guide including specific questions to guide the discussion (17) (Table 1). A non-directive approach to moderation was adopted. The discussions were tape-recorded and the recordings transcribed verbatim. The two facilitators debriefed immediately after each focus group to share their observations and to facilitate the recording of important details (18). One of the moderators and another researcher then transcribed and analyzed the data. The transcripts returned to participants for comment and/or correction, and participants provided feedback on the findings. The researchers discussed data saturation. Inductive content analysis of data was used (19). Each focus group transcript was considered as a unit of analysis. The analysis process included: open coding, category creation and abstraction phase. Two researchers independently read the transcripts and defined codes, categories and subcategories. A researcher expert in qualitative analysis confirmed the codes and categories.

**Table 1:** Topic guide.

1	Did your professional and personal motivation and the motivation of your colleagues change during the COVID-19 pandemic? If so, how? If so, what do you think is the reason for the change?
2	What is your experience with respect to the attention at the “person centered care” during the COVID-19 pandemic?
3	Has the experience of the COVID-19 pandemic had an impact on your personal and professional skills? If so, how? If so, why?
4	Can you tell me about your work experience as a nurse in a COVID Center?
5	Can you tell me about the main areas for improvement emerging during the COVID-19 pandemic? Could you tell me what you have learned from this experience?

## Results

Two focus groups were carried out, with a total of nine participants, including eight staff nurses and one nurse manager. Nurses' ages ranged from 28 to 46 years (mean age: 35.4 years; SD: 5.6); seven were female and two male. They worked in different wards before the experience in the COVID Center. Each focus group lasted from 60 to 80 min. None of the nurses had ever participated in a focus group. Participants were pleased to share their experience as front-line nurses in COVID Centers, and they were grateful for this opportunity, seen as an important moment to re-elaborate and reflect on their own lived. The discussions were characterized by empathy, involvement and respect for the experiences and opinions of other participants.

The findings can be summarized in five main categories: motivation and sense of mission; development of professional and personal skills; spirituality, feelings and emotions; person-centered care; uniqueness of the lived experience (Table 2).

**Table 2:** Themes and sub-themes.

**N**.	**Themes**	**Sub-themes**
1	Motivation and sense of mission	Professional identity, professional value and sense of duty
		Desire to participate in the battle against COVID-19
		Voluntary choice
		Camaraderie and sense of belonging to the professional group
2	Development of professional and personal skills	Teamwork and unity
		Personal growth and humility
		New skills acquisition
3	Spirituality, feelings and emotions	Prayer and God
		Mixed feelings
		Tiredness and fatigue
4	Person-centered care	Relationship between nurses and patients and patient-centered care
		Basic care and body care
		Centrality of professionals and role of hospital management
5	Uniqueness of the lived experience	Indescribable experience and a sense of isolation
		Inhumane experience for patients and families
		Like in a war

### Theme 1: Motivation and Sense of Mission

#### Professional Identity, Professional Value and Sense of Duty

Many of the nurses involved expressed a motivation also driven by a strong sense of mission linked to their professional identity, sense of duty and the desire to participate with their own weapons in the battle against COVID: “*The sense of duty has pushed me...” [N3]; “This war is not fought with weapons, the classic ones, it is fought with health care, so as a health worker it seemed normal to me... to offer my availability to try to help” [N3]; “… It's like having been called to enlist” [N3]*.

Together with a strong civic sense: “*... when it was proposed to me [to work in the COVID Center], a sense of duty prevailed, with respect of my profession, but also as a citizen and also in front of my children” [N2]*. This motivation also led to an explicit identity and professional pride, as a nurse said: “*Oh God, now I'm a nurse, now it's mine … I felt involved and called. [N4]”*.

#### Desire to Participate in the Battle Against COVID-19

Participants unanimously demonstrated a strengthening of professional motivation during the pandemic. For example, a nurse said: “*I was very determined to offer my availability because there was a need for nurses and I am a nurse, I am proud of being nurse and therefore it was right to try to do it.” [N3]*. This desire to participate has not faded over time: “*In my opinion, motivation has never been lacking …” [N1]*; “*I never thought of giving up.” [N8]*. Other words that emphasize the strong will that has characterized the nurses: “*One cannot fail to be in this moment.” [N4]*.

#### Voluntary Choice

The work of the nurses involved in the COVID Centers was characterized by the freedom of choice. The nurses were called, on a voluntary basis, to work with COVID patients, and the response was immediate: “*Immediately by instinct” [N3]*, “*I immediately decided to get off [in the COVID ward]”*. According to some of them: “*… the main motivation was the same that led me, years ago, to make this career choice.” [N1]*, representing a confirmation of the professional choice, which is also motivated by the desire to take care of persons. A nurse said: “*I chose a profession that somehow it was oriented to do something for others as well.” [N1]*. The issue of determination in the voluntary choice emerges several times by different nurses: “*My request.” [N7]*; “*It was my choice.” [N2]*; “*Voluntarily …” [N4]; “I asked to be moved to the COVID ICU.” [N8]*; “*I had no doubts, I'm going!” [N3]*. Those who were unable to participate in some moments even felt deprived of this possibility: “*I felt a little deprived of this great opportunity.” [N9]; “I resigned [from my previous job] to be able to work in the COVID Center.” [N8]*. From the words of the participants it is clear how the motivation remained stable: “*I have never lost motivation.” [N1]*, “*I never thought of giving up …” [N5]*, and even in some cases it seems to have increased: “*Motivation is back.” [N1]*. A nurse said: “*If today they asked me ‘there is another COVID center to open, there is another moment of pandemic, would you start again?' I would say yes again. It was one of the most professionally stimulating experiences.” [N3]*.

#### Camaraderie and Sense of Belonging to the Professional Group

A common theme noted by all participants was a particular sense of belonging to the professional group and the climate of continuous mutual support, so much that they noted: “*You become a family” [N7]*. These represent some of the main levers of motivation, as some nurses reported: “*We said: - oh thank goodness we come to work! Because we are among us, we are fine, there was a lot of complicity.” [N8]; “There was fear but there was also the energy and happiness of being in a good group.” [N7]; “The help of the group, because they were really close, they did a great job, together we did it.” [N1]*. This sense of belonging also had impact on personal lives of professionals. Nurses report experiencing a sense of belonging and “*That strange feeling of camaraderie …” [N8]* that made the work environment like a family: “*It wasn't a family, it wasn't a work group, it wasn't… it's something that goes beyond everything.” [N3]*. On the other side, a certain difficulty emerged in the relationships outside of work, as a sort of fear of not being included, in contrast to the workplace that becomes the comfort zone, the safe space in which to feel understood, despite the risks linked to the possibility of contagion. As claimed by a nurse: “*… now that you start meeting people who have a role outside... friends, relatives, who have nothing to do with your work, you don't feel comfortable with them* …” *[N7]*. Other participants shared: “*The others live it [this situation] just differently.” [N7]*; “*Colleagues are the only people who understand you.” [N7]; “Instead, your colleagues know what you are facing.” [N2]*; “*I only trust you …” [N1]*. Nurses seem to have more confidence, and feel more comfortable with the people with whom they have shared the COVID period, so much so as to lead a nurse to say: “*But … you know, maybe I will miss all this.” [N8]*.

### Theme 2: Development of Professional and Personal Skills

The participants considered their experience in the COVID Center as a “*great opportunity to get involved …” [N8]* as it was lived as “*… a professional and personal growth, first of all.” [N3]*. They shared that “*We all came out of there with an important training experience.” [N7]* and that “*The whole group has grown a lot.” [N5]*. Other representative statements are for instance: “*I really feel like another person.” [N6]* and “*An improvement of everything … everything.” [N1]*.

#### Teamwork and Unity

An experience of a great teamwork emerged from the discussions: “*Supporting each other … for me this was fundamental because not everyone can do everything. But not on a technical level … on a technical and practical level we are all capable... I mean … on a personal and emotional level.” [N6]*. The support concerns both, the technical-professional field, and the collaboration to address fear, fatigue, weight and difficulties characterizing the work in the COVID Centers.

A nurse said: “*There you reset everything … and everyone has brought their own contribution.” [N1]*. It is worth dwelling on the latter expression; indeed, many nurses found themselves working in new wards and with a type of patients never attended before. Everyone has used their skills to better cope with the emergency, as expressed by a nurse: “*Because we helped each other in everything…” [N4]*, an “everything” that goes from monitoring critical patients to managing of death or communication with patients and their family. They worked so that everyone put their skills at the service with the willingness to learn. As they noted: “*There was no one great, neither new, nor old, nor experienced, nor without experience, it was as if we all started from scratch.” [N3];* and also: “*We were all like in our first work experience.” [N1]*.

#### Personal Growth and Humility

Some nurses often used the terms *Humbleness* and *Teamwork* to explain the professional experience lived in the COVID Centers. One of them said “*… and humility … that if sometimes you have forgotten it, here it comes back strongly, and it is crucial as a person and as a professional” [N3];* another *One* stated that it was normal to say: “*Teach me because I don't know how to do it …” [N3]* or “*Guys, I can't do it with this patient now, come with me or do it for me, because I can't …” [N6]*. A nurse told the experienced atmosphere: “*COVID made us make a single team... we helped each other in everything.” [N7]*; “*They were really close to me …” [N2]*; “*We just couldn't do it alone.” [N7]*. A common theme emerging from participants was the great trust established in a very short time among colleagues who did not know each other before or had never worked together, accompanied by a growth in “*… a great self-confidence.” [N1]*.

#### New Skills Acquisition

In addition to the specific skills related to the use of Personal Protective Equipment (PPE), participants focused on the growth in advanced skills for critical care, such as: “*… positioning a non-invasive mechanical ventilation …” [N7]*. A nurse expert in the critical area, who guided less experienced staff, explained that “*At the beginning it was difficult, now they have learned to be quick, to receive instructions, to put them into practice and certainly also to make proposals, to feel part of that department.” [N3]*. Participants also reported the growth in problem solving and decision making skills, given the need to find and propose solutions quickly. It was common to “*… make decisions quickly.” [N1]* or “*… to manage situations you have never experienced before …” [N2]* as “*You had to face something that no one knew.” [N7]*. A nurse said: “*We have learned to be fast”. [N1]*.

### Theme 3: Spirituality, Feelings and Emotions

#### Prayer and God

Some nurses reported having rediscovered the meaning and the need to pray, as one of them said: “*I think I have never prayed as much as since I'm in the COVID Center.” [N6]*, and prayer has been a constant, thanks also to the presence of a Priest completely dedicated to COVID-19 patients and staff.

#### Mixed Feelings

It turned out that nurses often experienced mixed feelings. In fact, despite the tragic situation, they showed gratitude for the lived experience and for how it was managed: “*We were lucky.” [N8]*, “*It was joy… You know that you really did everything you could.” [N8]*; “*It was gratifying... Like those who manages to reach the top [of the mountain].” [N2]*; “*We are happy to have succeeded and I thank you for that time there.” [N3]*. At the same time, feelings of joy alternate with “*Uncertainty”*, “*Pessimism” [N2]* and “*Sadness” [N5]*, along with fear and anger. Fear is linked above all to the fear of contagion, for oneself but above all for the loved ones: “*You were afraid of having the virus... but the fear was mainly linked to the family.” [N1]*; “*I'm afraid... afraid of infecting someone.” [N9]*; “*I lived the first few days with fear… fear of being able to infect some relatives.” [N5]*, “*Fear to make a mistake … fear of hurting the people around you.” [N6]*.

#### Tiredness and Fatigue

Physical and emotional fatigue linked to the situation and also to the need to work with the PPE emerged from all the participants: “*In my opinion we almost feel a depression.” [N8]*; “*It really tested me on a psychological level.” [N3]*; “*It's been a terrible month.” [N2]*; “*It's no small thing to be in there.” [N6]*; “*You can't go to the bathroom, take a shower because you have the anxiety that they call you.” [N8]*. Some nurses said that in some moments they would have wanted to escape: “*I couldn't wait to go away…” [N5]* and “*Maybe it's the case that I get out of this place…” [N6]*; “*It takes away a lot of energy.” [N4]*; “*You were exhausted.” [N9]*. At the same time it emerged that, although nurses could ask to be moved, they did not; some of them stated that: “*The operator hardly says he is tired… it would be a bit like betraying other colleagues.” [N4]*.. Fatigue has also often been related to the use of PPE, as reported by some nurses: “*Obviously, fatigue increases because you have to do everything with PPE.” [N7]*.; “*You stay... hours inside the PPE… Completely foggy…” [N1]*.

### Theme 4: Person-Centered Care

One of the biggest challenges was, on the one hand, the communication between healthcare professionals and patients/family members, on the other hand the commitment to do as much as possible to foster communication between patients and their families, and to accompany the patients in such a difficult moment of their life also characterized by the absence of the affection and closeness of relatives.

#### Nurses-Patient Relationship and Patient-Centered Care

Despite the complexity of care, nurses rediscovered the importance and effective of some elements and behaviors of caring. Difficulties in the relationship due to the critical clinical conditions of patients, the presence of the PPE, and the absence of relatives, favored the establishment of highly significant relationships with patients in which even small gestures, acts of closeness and listening were rediscovered as an important part of care: “*The patient-centered care was one of the things on which... we worked a lot.” [N5]*; “*Patients also needs a chat, a word … a word of comfort.” [N1]*; “*We do things that maybe we didn't do before... more care also in terms of appearance... pay attention to these things, to the beard, to fix it... a caress every now and then...” [N7]*; “*A lot, but really a lot of attention …” [N7]*. Some nurses affirmed that they felt like the patient's family: “*The centrality of patients... as far as we operators are concerned, we also act as relatives …*” *[N9]*, another sums it up as follows: “*So you really put him at the center, in everything... We have put the patient at the center of everything” [N4]*. A nurse remembers the words of a patient: “*Ok, I trust you, you are my wife that I cannot have around.” [N6]*. Another said: “*At that moment you are objectively the only person next to them” [N5]*. All of the participants experienced significant care relationships with the patients and the development of a deep feeling of empathy: “*I think that I have never had a relationship like this with patients before …” [N6]*, which also concerned for example: “*giving some extra attention to a person who... maybe needed to be motivated...” [N8], “… [giving] an extra caress every now, when you can, when you can't - almost never - but when you can you do it” [N8]*. On the one hand, it was like rediscovering some patients' needs, such as that of esteem and belonging, which in ordinary conditions of care might remain in the shade. For example, a nurse noted that “*…you are objectively the only person next to them … if the patient asks for help for a video call with his relatives and you can't because of the workload, you realize that a video call for patient is a very important …, that does it matter, and you think: this patient is asking for a video call and I have too much to do to help him!” [N4]*.

#### Basic Care and Body Care

The nurses said they rediscovered the importance and effectiveness of basic care: “*It becomes important to cut patients' hair or shampoo them, which are things you can't do every day in the ordinary wards... Take a lot of care of patients' hygiene and body care.” [N3]*. Speaking about the patients' death, it emerged that “*The moment of death, yes it is hard …” [N6]*, “*You think that relatives will never see it … you see patients terrified … That is one of the particular moments that not everyone [nurses] are able to cope” [N6]*, and “*… an extreme personal care is needed... even in that extreme moment …” [N4]*. Speaking abut the feeling of helplessness associated with seeing patients to die alone, within the team they found the solution to give each dying patient as much as possible in basic care, to better prepare them for the moment of death: “*At a certain point... with my group... we talked... and we started a little to anticipate situations. When you know that the situation evolves in that way, then... in the end we hurry more to do things, which can be to wash that person well, take better care of their body, because you know that you have to... ‘send them away' like this... the feeling was somehow we lacked the dignity of the person... because the relatives will never see it... but you... you really did everything and... in short, we discussed among us and we found this solution.” [N1]*.

#### Centrality of Professionals and Role of Hospital Management

The nurses felt protected and cared for by the continuous and timely communications, provided by the hospital management, and by the resources provided to prevent the risk of contagion (PPE, availability of room and board for those who wished, protected paths for hygiene staff after the shift, etc.). Nurses reported that: “*Our hospital has also put us nurses and doctors, in short, all the staff who worked in COVID at the center of attention …” [N9]*; “*In terms of centrality of person, I did not feel abandoned” [N5]*; “*I don't know how many hospitals have given this opportunity and have treated their staff as we have been treated … we have been very supported … nothing has ever been lacking here” [N5]; “There was too much attention for us at the COVID Center*.” *[N1]*.

### Theme 6: Uniqueness of the Lived Experience

#### Indescribable Experience and Sense of Isolation

The uniqueness and indescribability of the lived experience was a common experience, together with the feeling of being understood only by those who have lived the same experience: “*Only those who have lived it can know” [N1]*; “*But am I only experiencing all this?” [N1]*. For some participants “*…there were no words to describe…” [N4]* and they experienced the common feeling of “*… managing something bigger than me*” *[N5]*. The sense of isolation also emerges, also described as “*… giving up a bit of normal life…”*, because “*You could not see friends, you avoided the family for fear…” [N8]*; “*…you avoid going to lunch, dinner, sharing your free time with others” [N2]*.

#### Inhumane Experience for Patients and Families

One of the themes perceived as more terrible and inhumane of the experience in the COVID Centers was seeing patients dying without the possibility of seeing family members, as a nurse shared: “*The fact of [absence of] the relatives, in my opinion, is against nature... because you think it is inhuman... It is not human for the patient and for those who stay at home waiting for the phone call once a day... It is not human that a daughter, a wife, cannot assist her husband, father or cannot see him for weeks or months” [N8]*. Another nurse stated that “*The lack of a relative is devastating for them” [N6]*, and it was moving to know that family members “*recorded the phone call to listen to it again to understand better.” [N8]*.

#### Like in a War

Participants expressed a shared idea, which was like experiencing a war: “*A feeling like being in a war” [N3]*. At the same time some nurses said that the experience was so unique that this war will probably miss them: “*Like the soldiers after the war, who are depressed because in the end they miss some of the war.” [N8]*.

## Discussion

This study aimed to explore the experience of nurses involved on the front line during the first period of pandemic. The main objective of the study was to explore whether and how caring for COVID patients had impacted nurses with a particular focus on professional motivation and the development of professional and personal skills. However, other important issues have emerged that are worth of discussion. The main themes found are: professional identity; motivation and sense of mission; development of professional and personal skills; spirituality, feelings and emotions; person-centered care; uniqueness of the lived experience. They shed new light on the correlation between motivation, professional identity, professional value, sense of duty and sense of belonging to the professional group; at the same time the experience on the front line in the COVID Centers represented a valuable opportunity for all the participants to rediscover specific aspects of nursing professional identity and to develop personal and technical-professional skills in a very short time.

A recent study (20) correlated the motivation of nurses engaged in the COVID emergency to the sense of duty and their career choice, and it reported how the pandemic has strengthened professional value. Nurses felt called into question, it was their moment, a mission for them, and they responded with courage, sense of responsibility and great sense of freedom, a freedom that is the ability to choose the good (21). Attention to emerging needs and the desire to contribute to the common good characterized the choice of nurses, which was immediate and voluntary. In line with other recent studies (9), the COVID experience made it possible to create strong and close-knit teams in a short time, and this had repercussions on professional motivation, and on the ability to deal with difficult situations, new work environments and need to learn advanced skills in a short time.

At the same time, the sense of belonging to the professional group, experienced as camaraderie, led nurses to a sort of self-isolation, whereby the reality in which they felt comfortable was precisely the workplace that became like his own family (22, 23). Nurses experienced the desire to be with their colleagues, by whom they felt understood, more than with relatives and friends outside the workplace.

Working conditions and the characteristics of the workplace strongly influence motivation (24). The sense of belonging to a team, a good working climate and support from colleagues have constituted a motivational drive to start the ‘adventure' in the COVID Center, and also to stay there as long as necessary (25). Nurses' professional motivation is therefore the result of the interaction between individuals, the workplace and the social context (26), and is above all intrinsic, an internal force that influences the person's behavior (27); in fact, none of the professionals related his motivation to external factors.

Nurses' experience with regard to the development of personal and technical-professional skills is very interesting. One of main findings is the great teamwork and the climate of union and continuous collaboration between everyone, regardless of the role held. Most nurses found themselves working in a new field and with colleagues they did not know, or with whom they had never worked before. It would be a great result to be able to bring back the lessons learned on teamwork within the COVID Centers even in ordinary care contexts. More experienced nurses joined less experienced ones; nurses with many years of experience were guided by younger nurses to learn how to manage critical patients. Everyone experienced and talked about humility and trust as virtues rediscovered and much appreciated through this experience.

Already the Framework of Disaster Nursing Competencies (28) highlighted the role that teamwork has in patient care in an emergency setting. Catania (29) in his study also highlighted the advantages of a good teamwork on the quality of care and on the well-being and mutual support of the staff involved on the front line. The study also confirms a growth in learning of skills such as problem solving and decision making, typical in situations of urgency and emergency. Consistent with recent studies (3, 5, 30, 31), the findings highlights that work-related factors impact positively on the development of fatigue, physical and psychological stress of nurses, often related to the use of PPE and to the increased workload. At the same time, the experience of conflicting and coexisting feelings emerges. In fact, despite the tragic situation, the nurses showed gratitude for the lived experience and for how it was managed, saying that “*… maybe we will miss all of this …”*. At the same time they experienced fear, anger and helplessness. In particular, similar to the findings of a recent studies (8, 32) fear among nurses has been mainly related and aggravated by being a possible carrier for family members.

An important result is the re-discovery by the nurses of the importance of some attitudes and behaviors which are essential part of caring, but which are sometimes overshadowed in ordinary care. In fact, the increasing difficulties in the relationship linked to the critical conditions of patients, the presence of the PPE, and the absence of family members, has led nurses to devote more time to relationships with patients. Small gestures, closeness, support in a video call, body care and listening have become an irreplaceable part of caring and have been rediscovered as such in all their value (33, 34); or to give more support than usual to other healthcare professionals, for example in communicating with a frail patient or his family, to obtain valid informed consent (35, 36). Nurses discovered in careful basic and body care the best way to provide dying patients all the necessary attention, and to be able to make up for the absence of loved ones as much as possible. At the same time, these attitudes have been a way for nurses to overcome the sense of helplessness experienced in numerous critical situations. Consistent with previous studies (8) our findings reported worries among nurses related to care provision. Sadness and worries are mainly attributed to witnessing patients' suffering and to the absence of their family (3, 32). Indeed, witnessing patients' sufferings, especially the end of a patient' life is one of the main sources of psychological pressure among nurses (37). In particular, during the pandemic, the absence of family of dying patients lead nurses to live this experience as ‘inhuman'. Being able to be cared for by those one loves is part of the dignity of the human person, and the nurses on the front line tried to take the place of the patients' families to give them the affection and dignity that every person deserves. For this reason it is important to promote the creation of systems and technologies that allow the family members to keep contact with the patients (38). Moreover, nurses can feel guilty because they can provide only limited care compared to usual care, for example due to the time required to wear PPE before contact with patients. These negative consequences can be associated with burnout, compassion fatigue and reduced well-being of nurses (39, 40). Improving psychological resilience is essential to cope with these issues (41, 42), and psychological counseling for nurses is recommended (32). Nurses' experience showed the key role of the hospital management in the protection and care of health workers, placing them at the center of the numerous and not easy decisions and policies taken during a pandemic (8).

Some limitations of our study should be acknowledged. Although generalizability is not a goal for qualitative studies, this was a single-center study conducted with a small number of participants who may not be representative of all nurses who worked in the COVID Centers during the COVID-19 pandemic.

## Conclusions

This qualitative study sheds new light on the experience of frontline nurses during the first period of SARS-CoV-2 pandemic. The findings are relevant for governments, managers, policy makers, nursing associations and healthcare organizations, in order to achieve continuous improvement in quality of patient care and in well-being and satisfaction of health professionals, even in ordinary situations.

The results underline the key role played by the work environment and by the group of colleagues in motivating and coping with physical and psychological fatigue due to care for patients in the COVID Centers (43). An effective support network includes the accompaniment, support and protection received by management, peers and co-workers in finding internal and external resources “*to take care of those who take care”*. An interesting relationship emerges between professional identity, sense of justice, basic care and professional well-being, and the importance of these elements for the delivering of excellent and effective care. Nurses rediscovered in careful body care and basic care-also and above all in critical area setting-an irreplaceable asset to restore dignity and to give the necessary closeness and attention to the often dying patient, also filling the absence of their familiars. These elements represent a way to concretely and deeply express the ethics of a Job Well Done. The study highlights how the need for esteem and belonging are essential elements of care, and that only love and benevolence are suitable and valid attitudes toward each person. For this reason, there is the need to actively engage new research, strategies and innovative technological tools, so that, even in situations similar to that experienced during the COVID-19 pandemic, a relationship can be maintained between patients and their loved ones.

Starting from the emerging findings, further studies are needed to deepen the main themes found involving a larger sample of nurses. In addition, performing a second study post-pandemic, and with a greater emotional distance from such a powerful experience, might lead to additional and novel findings.

Future research is needed more than ever to prepare healthcare for the future in similar situations, and a person-centered approach guided by an ethics of a Job Well Done will be the key to success.

## Data Availability Statement

The original contributions presented in the study are included in the article/supplementary materials, further inquiries can be directed to the corresponding author.

## Author Contributions

ADB: study design, writing, data curation, and analysis. RG and CP: revision. SS, AM, GF, AP, CD, and ID: data curation and analysis. MP: data curation, analysis and writing revision. FD, RA, and DT: conceptualization. All authors contributed to the article and approved the submitted version.

## Conflict of Interest

The authors declare that the research was conducted in the absence of any commercial or financial relationships that could be construed as a potential conflict of interest.

## Publisher's Note

All claims expressed in this article are solely those of the authors and do not necessarily represent those of their affiliated organizations, or those of the publisher, the editors and the reviewers. Any product that may be evaluated in this article, or claim that may be made by its manufacturer, is not guaranteed or endorsed by the publisher.
